# Meeting the brain on its own terms

**DOI:** 10.3389/fnhum.2014.00815

**Published:** 2014-10-13

**Authors:** Philipp Haueis

**Affiliations:** Max Planck Research Group “Neuroanatomy and Connectivity”, Max Planck Institute for Cognitive and Brain SciencesLeipzig, Germany

**Keywords:** cognitive hypotheses, concept formation, exploratory experiments, mesoscopic scale, connectivity

## Abstract

In contemporary human brain mapping, it is commonly assumed that the “mind is what the brain does”. Based on that assumption, task-based imaging studies of the last three decades measured differences in brain activity that are thought to reflect the exercise of human mental capacities (e.g., perception, attention, memory). With the advancement of resting state studies, tractography and graph theory in the last decade, however, it became possible to study human brain connectivity without relying on cognitive tasks or constructs. It therefore is currently an open question whether the assumption that “the mind is what the brain does” is an indispensable working hypothesis in human brain mapping. This paper argues that the hypothesis is, in fact, dispensable. If it is dropped, researchers can “meet the brain on its own terms” by searching for new, more adequate concepts to describe human brain organization. Neuroscientists can establish such concepts by conducting exploratory experiments that do not test particular cognitive hypotheses. The paper provides a systematic account of exploratory neuroscientific research that would allow researchers to form new concepts and formulate general principles of brain connectivity, and to combine connectivity studies with manipulation methods to identify neural entities in the brain. These research strategies would be most fruitful if applied to the mesoscopic scale of neuronal assemblies, since the organizational principles at this scale are currently largely unknown. This could help researchers to link microscopic and macroscopic evidence to provide a more comprehensive understanding of the human brain. The paper concludes by comparing this account of exploratory neuroscientific experiments to recent proposals for large-scale, discovery-based studies of human brain connectivity.

## Introduction

Since the wedding of cognitive psychology with positron emission tomography (PET) in the 1980’s and functional magnetic resonance imaging (fMRI) in the 1990’s, neuroscientists commonly assume that “the mind is what the brain does”. In the broad generality of a neuroscientific textbook opening, the assumption entails that “all of behavior and all of mental life have their origin in the structure and function of the nervous system” (Squire et al., 2012, p. xix). In functional neuroimaging—which will be the focus of this review—researchers therefore often ask participants to execute a task (e.g., finger-tapping, counting, memorizing numbers) while non-invasively measuring brain activity. Since the measured differences in activity are thought to reflect the exercise of human mental capacities, human brain function is primarily understood psychologically (Kosslyn, 1999). In the last decade, however, several methodological (resting state fMRI), technological (diffusion weighted imaging, i.e., DWI) and theoretical (graph network analysis) advances made it possible to study human brain connectivity without using cognitive tasks or constructs in the experimental design (Tournier et al., 2004; Shehzad et al., 2009; Sporns, 2011, 2014). The emerging field of connectomics combines such advances with classic methods (e.g., micro-anatomy and electrophysiology) to uncover organizational principles of the human brain. In contrast to hypothesis-driven, task-based research, the discovery science of connectomics often uses data-driven methods and large sample sizes (Sporns et al., 2005; Biswal et al., 2010). The term “connectome” furthermore puts the significance of connectivity approaches next to Big Science such as the Human Genome Project (for a review, see Lichtman and Sanes, 2008). But scientific criticism of their “uncognitive” nature often leads researchers to frame connectivity studies in psychological terms (e.g., “default mode network”), or to promise that their results will be relevant for the treatment of behavioral and psychiatric disorders (such as ADHD or Alzheimer). Given the current co-existence of both approaches, it seems an open question whether the working hypothesis “the mind is what the brain does” is indispensable in human brain mapping and its link to other methods such as electrophysiology.

My contention in this paper is to show that the hypothesis is, in fact, dispensable. Its maintenance prevents researchers from scrutinizing which concepts are adequate to describe the human brain. If the general hypothesis is dropped, researchers can “meet the brain on its own terms” by conducting exploratory experiments that do not require the testing of particular cognitive hypotheses. Such experiments would allow neuroscientists to search for new, non-cognitive concepts to describe the human brain. The first part of the paper introduces a theoretical framework that justifies the use of exploratory experiments by showing how neuroscience is methodologically autonomous from cognitive psychology. Such a framework is needed since common philosophical conceptions of neuroscience view the assumption that “the mind is what the brain does” as indispensable for investigating human brain function. The second and main part of the paper then puts this framework into practice by introducing three research strategies that do not require the use of cognitive concepts. By using historical examples of exploratory experiments in physics and molecular biology I show how such experiments may allow brain researchers: (i) to form new concepts and formulate general principles of brain connectivity; and (ii) to combine connectivity studies with manipulation methods to identify neuronal entities in the brain. I argue furthermore; that (iii) both these research strategies may be most fruitful if applied to the mesoscopic scale of neuronal assemblies, since the organizational principles at this scale are currently largely unknown. Based on this account of exploratory experimentation in neuroscience, the last part of the paper discusses to what extent large-scale proposals of discovery-based connectivity mappings could meet the human brain “on its own terms”.^1^

## Theoretical foundations: towards a new perspective on human brain organization

This paper starts from the assumption that the principles governing the structure and function of the brain can be asserted independently from the principles that make the capacity for human mentation intelligible. The reason for this independence is that many scientific practices disclose entities—such as genes, tectonic plates or spiral galaxies—for which there are little or no conceptual resources for describing them in non-scientific language (Rouse, 2011). Similarly, neuroscientific practice specifies the criteria for entities such as neurons, cortical areas and networks, for which there are little or no descriptive resources in our ordinary discourse about human mental capacities. Because brain researchers and philosophers of neuroscience commonly assume that “the mind is what the brain does”, it is often overlooked that the criteria for being a mind or a brain are developed and maintained in distinct practices.^2^ Emphasizing the difference between both practices resembles the argument that neuroscientists misuse psychological concepts, whose proper logical subject is the whole human being, by applying them to a part of such beings, i.e., the brain (Bennett and Hacker, 2003). Since proficient speakers of ordinary language do not have any conception of what it would be for a brain to think, decide or feel something, the attribution of such concepts to the brain makes no sense. Equivalently it does not make sense to apply the opposites of these concepts to the brain: “The brain neither sees, *nor is it blind—*just as sticks and stones are not awake, *but they are not asleep either*” (Bennett and Hacker, 2003, p. 72). The fallacy, in other words, consists in conflating two different sets of criteria in one statement, by describing what activity patterns are possible for neurons, synapses, areas or networks in terms of what behavior is possible for the whole human organism.

Although my practice-based view of neuroscience resembles Bennett and Hacker’s general critique that brain scientists often do not use psychological concepts properly, their argument is both too strong and too weak for the position advocated here. It is too strong if the logic of applying psychological concepts in everyday speech is taken as evidence for the claim that the “mind is *not* what the brain does”. In order to establish new concepts to describe the brain more adequately, researchers would neither have to affirm or deny any such claim. They would instead need a new perspective that would allow them to investigate the human brain without having to use cognitive vocabulary to describe its function. Bennett and Hacker’s position is too weak to explicate what kinds of research strategies would contribute to the establishment of such a perspective. Even if the activity of brain areas is not equated with mental capacities, they conclude that researchers should still “correlate neural events and processes” to psychological concepts, or identify the “*causally necessary* conditions for the human being to think or perceive, imagine and intend” (Bennett and Hacker, 2003, p. 117). On the basis of task-based fMRI studies, however, it is difficult to assess whether correlating psychological concepts to changes in neural activity adequately captures human brain organization. Part of the difficulty is that isolating cognitive components by subtracting a control condition from changes in neural activity while the subject executes a task is circular (Van Orden and Paap, 1997). It assumes not only that the actual cognitive components are known—and are accurately captured by concepts like “working memory”—but also that the components correspond one-to-one to the activated brain areas (modularity), which themselves are connected in a strictly feedforward fashion. Only then can it be ruled out that other activity changes do not influence the cognitive component that was localized in a particular brain region by means of subtraction. Given our current physiological knowledge of the fMRI signal, this feedforward view of brain organization is most likely to be false (see the section below on neural entities for further discussion). What is important for now is that the methods of task-based fMRI studies lead to the functional localization of alleged cognitive components regardless of whether the modularity and feedforward assumptions are true. Consider for instance, how statistical significance tests are used to determine which voxels contain neurons that were active during the subject’s execution of a task. Applying a conventionally statistical threshold (most commonly *α* = 0.05) to the recorded data will result in isolated activation patterns, even though the null hypothesis (i.e., that the experimental task had no effect on the data) is strictly speaking false for causally dense systems such as the brain, in which every neuron is at least weakly connected to every other neuron (cf. Savoy, 2001). Since the method of statistical thresholding arbitrarily excludes small, but potentially task-related changes in neuronal activity from activation maps, these maps do not provide proper evidence for localization hypotheses that predict “that a given brain region plays a particular causal role during the performance of a cognitive task” (Klein, 2010, p. 265).

This short review of the issues of subtraction and statistical testing does not imply that the use of cognitive tasks and constructs in fMRI studies is altogether unjustified, especially since more sophisticated experimental designs and statistical analyses exist (Klein, 2010; Poldrack, 2010). Nor does it suggest that other approaches such as anatomical or functional connectivity studies are exempt from issues such as arbitrary thresholds, for instance when cortical areas are defined by a family of clusters with similar connectional fingerprints (Passingham et al., 2002). It rather points out that in order to eventually use cognitive concepts in a noncircular way, neuroscientists would first need an independent check on whether the activation patterns found through task-based studies adequately reflect an aspect of human brain organization.^3^ Such a check could be made possible through a perspective on human brain function and organization that does not proceed by a hypothesis-driven search for the causally necessary conditions of mental capacities such as “memory”, “attention” or “perception”. The data-driven methods of connectomics—so I shall argue—might provide or contribute to such a perspective, and therefore seem to be the most promising candidate to meet the brain “on its own terms” to date. Neuroscientists have investigated the anatomical or chemical structures of the human brain, as well as its metabolic or homeostatic functions without using cognitive concepts, long before connectomics emerged as a separate subdiscipline. But what seems to be distinctive about the perspective of connectomics is that it provides general principles (such as network motifs that specify recurrent “building blocks” within complex connectivity patterns cf. Sporns and Kötter, 2004; see also Song et al., 2005) that reliably track known functional and structural features of human brain organization (cf. Shehzad et al., 2009; Tymofiyeva et al., 2014). Given this convergence with former anatomical and physiological evidence (but also evidence from task-based studies, cf. Nelson et al., 2010), connectomics researchers are now in a position to develop new research strategies which do not presuppose a psychological theory or cognitive hypotheses that decompose human brain functions into cognitive components. The target of such research strategies would be the extension of connectomics studies to the level of “working principles”. Such principles specify how brain parts defined by their connectivity interact with one another, given what is known about the anatomical, chemical and metabolic constraints on human brain function. Ideally, understanding the brain in terms of connectivity would also elucidate how brain networks interact with the rest of the body so that whole human organisms exhibit intelligent behavior (Sporns, 2011). Notice that as a result of the independence from cognitive, hypothesis-driven approaches, it could even be possible that these new connectomics research strategies lead to results that are *incompatible* with our previous understanding of certain interactions of neurons, areas and networks as “causally necessary conditions” for mental capacities.^4^ Whether they are incompatible will depend on the results established through the types of exploratory research strategies that are discussed below.

Even if connectomics represents a new perspective on human brain organization, it still needs to be shown *why* the corresponding research strategies would not employ cognitive concepts in neuroscientific practice. Consider therefore how a general psychological statement about human brain function would be confirmed by an experimental neuroscientific result. Visual perception experiments, for instance, indicate that action potentials directly invoked through eye stimulation are accompanied by many signals from areas whose function is to detect more specialized features. The ratio between signals from the eye and from upstream areas suggests that perception involves comparative processes between present and past stimuli. But the forms of neuronal adaptive activity that make such comparisons possible are the *same* as for other phenomena such as learning or memory (Wolpert, 2007). By reference to the same neuronal adaptive activity these cognitive phenomena become empirically indistinguishable from one another. The flipside of the same problem is that a diverse group of neuronal processes can account for the *same* psychological construct, depending how it is functionally decomposed (see the example of “intention” in Turner, 2012). In order to distinguish—or in the intention case, unify—cognitive behavior on the basis of neuroscientific evidence, researchers additionally need to consider what patterns in the data are made *salient* from a certain *conceptual outlook* (Lange, 2000b). Compare how in chemistry, the Van der Waals gas law makes a shift from an increase to a decrease of the product of pressure and volume salient, when researchers add the “working assumption that all gases are composed of small ceaselessly moving particles attracting each other by short range forces” to the mathematical equation (cf. ibid., 219). From this “molecular gas” outlook, gas particles will be so close together at high pressures that the volume will decrease slower than the pressure will rise. In a similar fashion, a psychological conceptual outlook on human brain function allows researchers to identify a salient pattern among the diverse neuronal processes that instantiate a construct like “intention”, or gives them additional cognitive behavioral evidence that distinguishes the outcome of neuronal adaptive activity into instances of “perception”, “memory”, or “learning”. The relevant parallel between the general statements of chemistry and psychology is that their empirical counterparts do only count *as* counterparts if the varying instances are subsume under the chemical or psychological concepts.^5^ Therefore, a perspective such as connectomics can only *be* new and independent from a psychological outlook if researchers do not evaluate divergent findings against the same salient patterns. Equivalently, the alternative research strategies within that perspective would only be new and independent if they employ alternative concepts, which allow neuroscientists to make salient generalizations about the activity patterns of neural entities that could be unintelligible from a psychological outlook.

The argument for an alternative, noncognitive conceptual outlook adds to common philosophical conceptions (e.g., Fodor, 1974) that *neuroscience* is autonomous from psychology. While the generalizations of both disciplines (i.e., their laws) often converge on what is actually happening, they can diverge in specifying what neural activity or psychological behavior is *possible*. Neuroscientific laws that specify possible patterns of brain activity could therefore hold even if psychological laws are *violated* (cf. Lange, 2000a,b). Consider Hebb’s law that relates the efficiency of signal transmission and temporally co-occuring pre- and post-synaptic activity. Casually stated as “neurons that fire together, wire together”, it would hold even if a change in natural and cultural evolution had resulted in humans with very different cognitive abilities. While generalizations like psychophysical laws about our perceptual capacities would be violated in this scenario, Hebb’s law remains stable—even if those brains are wired differently—because it only specifies *when* the strengthening of neuronal connections is possible or impossible, not *what* specific connections will form in the brain. In fact, Hebb’s law could even hold if these brains are wired *similarly* to ours, while their bearers possess different cognitive functions. The reason is that the role of a specific (neural) mechanism depends on its integration into the functional economy of the whole organism (i.e., how an organism responds to and interacts with environmental pressures or cues within its environmental niche). In genetics, the *Pax6* gene is highly conserved across species (humans, mice, *Drosophila*) in the mechanism of eye development, but the resulting light-sensing organs differ widely as their role differs across species-specific navigational patterns, food-searching behavior or visual discrimination of environmental cues (Carroll et al., 2005). With respect to the evolutionary distinct version of *homo sapiens*, it is similarly possible that they share with us the connectivity patterns formed by Hebbian synaptic strengthening. The functional role of these patterns, however, would differ with respect to which evolutionary pressures these organisms had to adapt to.

To summarize, in this section I introduced a theoretical framework to argue for a new perspective on human brain organization from which researchers can specify the criteria for neural entities, without having to assume which causal role these entities play in cognitive processes observed at the behavioral level of human subjects. Specifying these criteria requires an alternative, non-cognitive conceptual outlook that allows an intelligible grouping of experimental results under general principles. The methodological advances of connectomics could provide such an outlook, and will be therefore discussed in more detail below. The example of Hebb’s law showed that such alternative generalizations could even violate psychological generalizations, and thus be unintelligible from our current cognitive outlook on human brain function. Note that similar conflicts could also occur in apparently non-cognitive aspects of neuroscientific practice such as anatomical classification. Color-opponent neurons, for instance, are functionally defined with regard to their opposite responses to colors that cannot be perceived in combination (such as green and red, cf. Zeki, 1993). With organizational principles such as connectivity rules, researchers could group these cells together with other anatomical elements, although they would not have anything in common according to the psychological theory of color-opponency. In order to search for non-cognitive concepts that enable such new inductive strategies, researchers would have to temporarily *suspend*, but not to eliminate psychological concepts (pace Churchland, 1989 and Bickle, 2003). But in the absence of a psychological outlook, such strategies would also not be “conceptually parasitic on higher-level theory” (pace Gold and Stoljar, 1999, p. 823). The psychological outlook may therefore be re-evaluated in the light of new results that were established through non-cognitive research strategies. The theoretical framework outlined above provides a third alternative to reductionist and non-reductionist positions in philosophy of neuroscience, because it views *both* neuroscience and cognitive psychology as methodologically autonomous from one another. The main part of this paper now attempts to show how this theoretical view makes a difference to neuroscientific practice, because it results in alternative strategies to investigate the human brain.

## Exploratory experiments in neuroscience

When scientists conduct exploratory experiments, their research is not driven by specific hypotheses or general theories, but rather by the attempt to systematically map out different aspects of the phenomenon under investigation (Hacking, 1983). Such experiments could meet the brain “on its own terms” because they would not require neuroscientists to test specific cognitive hypotheses about human brain function. Resulting from a one-sided focus on theory and hypothesis testing, philosophers of science and scientific methodologists have repeatedly criticized exploratory research as “blind data gathering” (in the case of neuroscience, see Churchland, 1989; Kosslyn, 1999). Such a criticism overlooks that the systematic character of exploratory experiments contributes to the *formation* of new scientific concepts. Without the most basic empirical concepts in a domain of inquiry—e.g., “chemical reaction”, “mechanical force” or “species”—descriptive or explanatory efforts of a scientific practice are not even conceivable. Because the disclosing function of scientific concepts is more fundamental than the theories that can be formulated with them, changing a conceptual framework can structure a whole field of later inquiry (Steinle, 1997). This section therefore uses historical examples of exploratory research to show how neuroscientists could develop new research strategies to investigate the human brain without cognitive concepts or hypotheses.

### New concepts to explore the brain? Lessons from electromagnetism

Early electromagnetic research provides an exceptionally clear historical case of how physicists developed new concepts by conducting exploratory experiments. In 1820, Christian Oersted made the surprising discovery that the connecting wire of a voltaic battery has an effect on a magnetic needle close by. From the conceptual outlook of physics at the time, the interaction between magnet and battery appeared unintelligible because electricity and magnetism were understood to be two separate physical phenomena. André-Marie Ampère investigated Oersted’s strange phenomenon by developing a new instrument, the “astatic needle”, whose axis is identical to the direction of the magnetic dip, thereby reducing the effect of terrestrial magnetism on the experimental apparatus. Having thereby isolated the phenomenon of interest, Ampère began mapping out its different aspects by systematically varying a large number of experimental parameters (e.g., strength and polarity of the battery, length and material of the needle). In order to describe the various effects of these alterations, Ampère had to reconceive existing concepts, such as “current”, and introduce new ones, such as the “single circular circuit” formed by the needle and the connecting wire (Steinle, 2002).

The example of Ampère’s research points out that exploratory experiments consist in the systematic, one-by-one *variation of experimental parameters*. The systematic variation then allows scientists to establish *empirically stable patterns* about the activity or behavior of the entities in the investigated phenomenon. Ampère’s combination of the astatic needle with the concept of the “singular circular circuit” also shows that in scientific practice, concept formation and technological development go hand in hand. Without properly working instruments, the phenomenon cannot be isolated from other, currently irrelevant patterns of the investigated system (i.e., the “noise”). But without the appropriate concepts, the measurement outcomes cannot be made intelligible as providing evidence for the activity or behavior of the entities in the domain. A successful series of exploratory experiments also identifies the parameters that are *necessary* for the production of the investigated effect (Ampère called this *fait general*, a “simple case” which displays a general pattern with particular clarity cf. Steinle, 2002). But previously successful experiments are not themselves sufficient for applying empirical concepts, because such concepts must also be projectible to hitherto unexamined cases (they have an “open texture”, cf. Waismann, 1945; see also Wilson, 2006). Similarly, the laws formulated with empirical concepts do not only reliably track previous empirical regularities, but also provide inferential norms for further experimentation. Such norms allow scientists to assess what unobserved instances would fall under a law’s scope based on a limited number of observations (Lange, 2000a).

The electromagnetism case provides the context for explicating the first research strategy with which researchers can meet the “brain on its own terms”, namely by developing non-cognitive concepts and formulating general principles of brain organization. Resting state functional connectivity studies are particularly suited for such exploratory research, because they allow researchers to vary a large number of experimental parameters. “Functional connectivity” can be experimentally determined using various instruments (PET, fMRI, multi-unit recordings, electro- and magnetoencephalography, i.e., EEG and MEG), resulting in different spatial and temporal resolutions as well as to whether connectivity is assumed between single-neuron activity, neuronal assemblies or large-scale neural interactions. Furthermore, there are multiple ways in which experimental parameters can be correlated (across or within conditions, subjects, or time courses). The absence of an explicit cognitive task in resting state studies that would drive the data analysis functions like the alignment of axis and magnetic dip in Ampère’s astatic needle experiments. It isolates the phenomenon of interest and the (potentially law-like) pattern it displays, namely slow, low-frequency fluctuations within the time-series recorded by the respective measuring instrument (such as hemodynamic responses in fMRI, cf. Biswal et al., 1995). From the psychological outlook of task-based experiments, such fluctuations were ruled out as unintelligible noise against the differential changes in activity that could be attributed to the subject’s execution of a task (although the fluctuations were already recognized in early fMRI studies, cf. Weisskoff et al., 1993). With the emergence of the resting state paradigm, such fluctuations not induced by cognitive tasks were reconsidered as neuroscientifically meaningful, because their temporal correlations are taken to reflect the statistical history of co-activated areas that are *functionally connected* to one another (Cohen et al., 2008).

It is important to note that resting-state and task-based analyses cannot be exhaustively contrasted along the task/non-task dimension. Resting state paradigms obviously include instructions (the subject is asked to remain still in the scanner), but importantly, there is no contrast condition that constrains how the recorded time-series are subsequently correlated to one another. What furthermore distinguishes resting state from “classical” task-based approaches is the theoretical shift to consider large-scale networks, rather than isolated functional areas as the analytical unit of functional architecture (Sporns, 2011). Data-driven analysis methods can also be combined with “unconstrained” experimental stimulations such as the free viewing of a movie (cf. Bartels and Zeki, 2005), suggesting that not all task-based studies are driven by cognitive hypotheses. Instead of a simple task/non-task dichotomy, the variety of neuroimaging methods may be therefore better captured by a combinatorial matrix with multiple dimensions (e.g., cognitive/non-cognitive, hypothesis/data-driven). While this paper focuses on exploratory experiments that do not employ cognitive concepts, analyzing other combinations of neuroimaging methods is an exciting task for further research (see also Dumit, 2014 in this special issue for a historical example of an exploratory neuroscientific experiment).

The possibility of establishing functional connectivity with various instruments and correlational techniques also marks an important difference to the electromagnetism case. Researchers have reached a consensus by defining “functional connectivity” as a “temporal correlation between spatially remote neurophysiological events” (Friston et al., 1993; quote from Friston, 1994, 58). But nobody has so far determined which experimental parameters are *necessary* for the production of activity patterns that count as functionally connected. While some commentators therefore criticized the concept as elusive (Horwitz, 2003), I argued elsewhere that “functional connectivity” is best conceived as *combinatorially vague*. Combinatory vagueness arises here because it is not only unclear how spatially remote two events have to be, but also because different neuronal events can fulfill the definition (Haueis, 2012). Ampère’s experimental series consisted in a one-dimensional measurement of electro-magnetic induction with one instrument, the astatic needle. In contrast, each imaging modality and analysis step has certain advantages over others for the investigation of functional connectivity, because the entities within its application domain are heterogeneous and complex. It may therefore be unlikely that the experimental system investigating the different aspects of the general phenomenon of functional connectivity can be “reduced” to a “simple case”.

In absence of a “simple case”, the status of general laws that govern all studies of functional connectivity has to be approached with caution. In a recent review, for instance, Palva and Palva (2012) proposed that spontaneous, infra-slow fluctuations (0.01–0.1 Hz) in EEG, fMRI and psychophysical measurements all point to the same underlying phenomenon, namely that the slow activity of large-scale intrinsic connectivity networks modulates how the activity of neurons and neuronal assemblies oscillates at higher frequencies. These oscillations in turn indicate the speed and strength with which electrical signals get transferred within the cortex. The evidence for convergence was that the infra-slow fluctuations in EEG, fMRI and psychophysical data could all be mathematically described by a scale-free power law, even though different mechanisms might underlie the oscillations observed at a specific spatial scale. Researchers would then be able to predict the pattern from the data of each modality with the same inductive strategy—i.e., that each time a scale-free, slow fluctuation changes its phase, the speed of the oscillation at a specific scale will change as well. The issue is that without necessary conditions for functional connectivity, similar mathematical properties of various measurement signals do not entitle neuroscientists to attribute all fluctuations to the same neural entity (i.e., large-scale, intrinsically connected networks). Based on the current technology, it is not possible to tell whether there are other neuronal assemblies whose infra-slow activity patterns require a different inductive strategy.

### How to identify neural entities with exploratory experiments

The comparison of connectivity studies to the electromagnetism case showed how exploratory experiments can help researchers to formulate general principles about human brain organization. But an example from experimental physics is insufficient to understand how entities subsumed under such principles are identified in multi-disciplinary contexts such as human brain mapping. This section therefore introduces an example from interdisciplinary molecular biology to examine why the use of cognitive tasks has left researchers uncertain about which entities underlie the changes in fMRI signal, and which alternative research strategy could help them to identify such entities via various experimental methods. Historical research on molecular biology has shown that different manipulation techniques can identify the same entities even if researchers hold conflicting theoretical commitments. The biochemist J. Monod viewed the genome as static (except for mutation) while the cellular bacteriophage geneticist F. Jacob viewed it as dynamic. By combining Monod’s temporal analysis of lactose digestion in *E. coli* and Jacob’s spatial DNA injection techniques, their collaborative experiments showed that enzyme gene expression followed immediately after DNA was injected in an inhibitor-free cytoplasm. Despite favoring Jacob’s view of the genome, the agreement upon the same entities and processes allowed both researchers to ask new questions about scientifically significant topics, such as the mechanism of protein synthesis (Burian, 2001).

The Jacob-Monod experiments resolved a theoretical conflict because they fulfilled the *mutual* *manipulability criteria* for mechanistic explanations (Craver, 2007). These criteria hold that an acting entity is a part of a mechanism if its activity changes once the whole system is manipulated, and that the activity of the whole system changes once a particular entity in it is manipulated. Only the combination of Monod’s and Jacob’s techniques fulfilled these criteria because “Monod did not have tools for *manipulating* DNA, for mapping genes, and for controlling crosses between bacteria, while Jacob did not have a *system* subject to the fine-grained controls that Monod did” (Burian, 2001, p. 395, emphasis added). For Craver, neuroscientific experiments that study phenomena like the action potential or the spatial orientation of rats in a water maze fulfill these criteria because they track causal structures trough activation, interference and intervention techniques (Craver, 2002). But none of these examples includes straightforwardly *cognitive* phenomena or technologies that are primarily used to investigate the *human* brain, such as task-based fMRI. Here, the working assumption behind the functional localization of cognitive tasks is that the BOLD contrast is an indexical trace of task-induced neuronal activity. But changes in fMRI signal do not only track spiking rates of single cells, but also neuromodulatory effects and feedback loops within cortico-cortical microcircuits. Because different oxygenation levels alone are insufficient to conclude whether the activity within these circuits is largely inhibitory, excitatory or whether inhibition and excitation balance each other out, the BOLD signal is *causally ambiguous* (cf. Logothetis, 2008). A task-related BOLD-increase is therefore not necessarily equivalent to a net increase in firing output of stimulus-specific neurons within the measured cortical area. Because the BOLD contrast indistinguishably subsumes this option among other physiological scenarios, fMRI technology alone does not provide the technical means to disambiguate the causal structure of excitatory-inhibitory microcircuits. Furthermore, the large population view of neuroimaging—where a voxel contains ~5 million neurons—prevents the assignment of “activity” to specific entities—be it circuits or single cells—that would be parts of a mechanism underlying the cognitive phenomenon.

Because fMRI evidence for task-induced changes in neural activity rests on a causally ambiguous physiological origin, fMRI experiments in cognitive neuroscience fail to meet the mutual manipulability criteria at the component level. Measuring system-level changes induced by cognitive tasks does therefore not decompose the organization of cortico-cortical microcircuits into its different active components such as excitatory pyramidal cells or inhibitory basket cells. Philosophers of neuroscience such as William Bechtel have previously underestimated the problem of the physiological origin of fMRI signal and concluded that “neuroimaging has produced many determinate results that have been incorporated into information processing models of cognition” (Bechtel, in press, p. 15). Bechtel proposes that the causal ambiguity of fMRI parallels the use of Golgi staining in neuroanatomy, where it is still unknown why silver nitrate only stains 1–5% of the cells in the prepared nervous tissue. But this parallel only holds for the *physical* assumptions behind fMRI. Only the magnetic properties of different cerebral tissue types are independent from the investigated domain in the same way as the chemical properties exploited to reveal neuroanatomical cell properties are.^6^ In contrast, the *biological* assumptions of cognitive scientists (i.e., that a task-induced BOLD increase reflects increased neuronal spiking output) are in direct conflict with the physiological knowledge about the organization of excitatory-inhibitory micro-circuits. But because cognitive neuroscientists do not provide a controllable system comparable to Monod’s in the molecular biology example above, these conflicting theoretical commitments could so far not be resolved by combining cognitive and neuroscientific methods.

If neuroscientists seek to explore the brain via various experimental methods, they would first need to establish which neuroscientific measurement techniques are able to identify which neural entities and activities. Such an exploration can proceed independently of whether such entities are causal preconditions for cognitive capacities. Noninvasive techniques (e.g., PET, DWI, EEG/MEG, fMRI) usually investigate activity patterns of single cortical areas or inter-areal networks, but their restriction to correlational techniques makes them insufficient to establish causal knowledge (for a critical review of methods that estimate causality in fMRI data, see Smith et al., 2011 and Lohmann et al., 2012). Here, technological innovation is required to address these currently unanswerable issues.^7^ While invasive electrode recordings can trace spiking rates from well-isolated neurons near the electrode tip, multi-unit recordings suffer from the same causal ambiguity as the BOLD-signal, because changes in the local field potential (LFP) are the sum of postsynaptic potentials, soma-dendritic spike after-potentials, and membrane oscillations of the surrounding neuronal assembly (Logothetis, 2008). Thus, no experimental technique so far unambiguously measures or manipulates the excitatory-inhibitory activity of cortical microcircuits, or the unimodal processing within cortical columns (Horton and Adams, 2005).

### Why we need precise exploratory experiments at the mesoscopic scale

This section introduces the parameters *scale* and *precision* in order to show the link between the issues discussed in the last two sections, i.e., between the missing necessary conditions for “functional connectivity” and the difficulties of manipulating neuronal assemblies. For the present purposes, a tripartite distinction of *scales* can adequately reflect the investigation of different aspects of brain organization in neuroscientific practice (Sporns, 2011). At the top of this scheme is the *macroscopic* *scale*, consisting of entities such as subcortical and cortical areas (e.g., hippocampus or orbitofrontal cortex) and large-scale inter-areal networks (e.g., the somatosensory-somatomotor network in the macaque cortex, cf. Honey et al., 2007). In the human case, the structure and function of such entities is usually investigated by noninvasive imaging modalities (fMRI, PET, DWI, MEG/EEG). At the intermediate stage is the *mesoscopic scale* of *neuronal assemblies*, consisting of entities such as inhibitory-excitatory micro-circuits or cortical mini- and macro-columns (Buxhoeveden and Casanova, 2002; Logothetis, 2008) that encompass populations between few 100–10,000 neurons. Below such neuronal assemblies is the *microscopic scale* of single cells such as neurons, glia and subcellular parts such as axons and synapses. Microscopic entities are usually the target of micro-electrode recordings or various tracer techniques in animal studies.

The second parameter, precision, consists of two subclasses, the first being the *degree of precision*, i.e., the (material) capacity of an imaging modality to display two spatially close measurement points within the brain as two different image points (e.g., pixel, voxel) in a brain map (i.e., its spatial resolution), or the capacity of an invasive instrument (e.g., electrodes, scalpels, chemical tracers, staining techniques) to allow structural or functional differentiation between different parts of nervous tissue. Equally, different instruments possess high or low temporal resolutions (Fingelkurts et al., 2005). The *actual precision* consists in the researchers’ ability to practically discriminate between different neural entities by *using* such invasive instruments or using what is displayed by brain maps (e.g., anatomical boundaries) in an experimental intervention (for a similar treatment of the precision of semantic terms, see Keil, 2010). The normative demand in neuroscientific practice is to match scale and precision in a way that the experimental conditions which are the criteria for applying empirical concepts can *account* for the entities (or their behavior) subsumed under such concepts.

With the parameters of scale and precision in place, it becomes clear that the issues discussed in the previous sections concern our current inability to intervene in and understand the mesoscopic scale of human brain organization.^8^ With regard to the power-law governed infra-slow fluctuations discussed in section “New concepts to explore the brain? Lessons from electromagnetism” it is unclear whether the extension of this principle to the activity of neuronal assemblies in between cellular mechanisms and macroscopic BOLD and EEG oscillations is warranted. If it was warranted, restricting it to micro- and macroscales would be an artificial limitation (similar to “All coppers in North America are conductors” cf. Lange, 2000a, p. 242). If it was not warranted, such an extension would be an unmotivated bend, because researchers would need a different inductive strategy to understand the functional connectivity of neuronal assemblies. Our current inability to resolve the indeterminacy between these two options results from the missing experimental techniques at the mesoscopic scale discussed in section “How to identify neural entities with exploratory experiments”. What neuroscientists would need is a *mesoscope*, i.e., an instrument (or a set of instruments) that can be used to measure or manipulate entities at the mesoscopic scale in a controlled and unambiguous fashion.^9^ The mesoscope is the presupposition for systematically varying experimental parameters, which in turn establishes the criteria for applying empirical concepts to the mesoscopic scale of the brain. Consequently, a successful exploratory experiment at the mesoscale would fulfill the criteria for mutual manipulation only with the degree of precision with which it describes an investigated brain mechanism. The mechanism of the action potential, for instance, can be described in coarse terms such as hyper- and depolarization, sodium and potassium channels, or in terms of sodium-voltage gated channels Nav1.1, Nav1.6 specific to human Purkjine cells (cf. Sirtes, 2010). But choosing the appropriate descriptive scope also depends on whether the material precision of the measuring instrument can be matched to the scale of the entities investigated in practice. Because of the causal ambiguity of LFP signals measured by multi-unit recordings, current invasive measurements do not satisfy the mutual manipulability criteria *with respect to* the mesoscopic entities (e.g., inhibitory-excitatory micro-circuits) for which such measurements are supposed to be evidence for. Given this scale-dependent failure of mutual manipulation, it is so far unclear whether the use of concepts like “cortical column” or “microcircuit” can establish determinate truth claims about the alleged entities these concepts are referring to (Horton and Adams, 2005; Logothetis, 2008).

The missing understanding of the mesoscopic scale also points towards the initial motivating factor for the need of exploratory experiments: the lack of a unified theory of brain function. Since both philosophers (e.g., Craver, 2007) and neuroscientists (see for instance http://www.humanbrainproject.eu/neuroscience.html) are critical of the idea that a *single* unified theory, if possible, is *necessary* to adequately understand the brain, the following argument will be put in the conditional form: *If* there *was* such a theory, it would have to provide both general principles for and the evidential relationships in between the three scales, in order to be explanatory. Without properly understanding how the uncharted territory of the mesoscopic scale looks like, the formulation of such principles and relationships is not possible or incomplete. Thus, exploratory experiments that target the mesoscale, together with the exploratory investigation of non-cognitive parameters at the micro- and macroscopic scale, will allow researchers to establish new and revise old concepts in order to meet the brain “on its own terms”. And *even if* it was the case that the laws or principles that practitioners are able to express with these concepts cannot be used to form *one* unified theory (such as in the case of electromagnetism), exploratory experiments will still increase our understanding since the patterns picked out by such laws would not only be independently stable, but perhaps also unintelligible from a psychological conceptual outlook.

## Exploratory experiments and connectomics research

With this scheme of the status and prospects of exploratory neuroscientific experiments in place, it is possible to assess the relationship between current proposals for large-scale mappings of the human connectome and the notion of “meeting the brain on its own terms”. What needs to be addressed first is whether the use of whole-brain imaging instruments (fMRI, DWI) in connectomics is suited for exploratory experimentation. The brief discussion in the section “Theoretical Foundations” suggested that hypothesis-driven fMRI approaches suffer from the weakness of statistical null hypothesis testing in causally dense systems such as the brain. Functional magnetic resonance imaging would be an appropriate instrument to test hypotheses if it belongs to the class of *narrow* instruments (such as a thermometer), which measure a small number of data points along one dimension (cf. Franklin, 2005). Functional magnetic resonance imaging experiments, however, record a large number of four-dimensional data points (time series per three-dimensional voxel): each recorded brain slice consists of thousands of voxels for each of which hundreds or thousands of measurements throughout the time of an experimental session are conducted. Functional magnetic resonance imaging also has a multi-dimensional signal: besides tracking the multifaceted oxy-deoxy ratio (consisting of cerebral blood flow, volume, metabolic rate of oxygen and glucose), it records “noise” (such as respiration rate or heartbeat) which can itself be subject to meaningful physiological investigation (Jezzard et al., 1993; Bianciardi et al., 2009). Taken together with the causal density of the brain, the large amount of multidimensional data suggests that fMRI—and by extension similar noninvasive whole brain imaging techniques—rather resembles *wide* instruments like DNA microarrays. Such instruments monitor the responses of a system as a whole, rather than recording the interactions of an isolated part within that system.

The above analysis, if correct, suggests that fMRI is indeed better suited for exploring system-wide interactions than to test cognitive hypotheses about isolated parts of neuronal interaction. Using such hypotheses in neuroimaging leaves room for exploration because they are so general that fMRI studies testing them leave open many possible outcomes (cf. Franklin, 2005). The reason for that openness is that employing arbitrary thresholds to study the causally dense system of the human brain does not entitle neuroscientists to infer that the activation patterns extracted from the data would have occurred *only* when the allegedly “confirmed” hypothesis was true (Klein, 2010). This lack of explanatory power is dramatically increased by the missing biological knowledge and manipulability of mesoscopic entities in the human brain. The analysis of whole brain imaging as wide instrumentation therefore suggests that while neuroscientists are *de facto* using general cognitive hypotheses to investigate the unknown aspects of the human brain, what is also at stake in such inquiries is how various *non-cognitive* experimental parameters are systematically related to each other in this causally dense system. The increasing recognition among neuroscientists that resting-state fMRI and other imaging modalities (e.g., DWI) can be used to map out “wiring diagrams” of the human brain (Biswal et al., 2010) reflects a partial recalibration of disciplinary interests to account for these non-cognitive parameters.

As has been shown in the case of the complete mapping of the nervous system of the nematode *C. elegans*, the establishment of wiring diagrams also represents a variety of exploratory research (Ankeny, 2000). Such diagrams of the canonical neural circuitry of an organism provide descriptive models, based on which specific hypotheses, theories and explanations can be subsequently formulated, developed and tested. Nevertheless, the construction of the *C. elegans* diagram relied on background assumptions such as the one-to-one correspondence between neuronal connectivity and the spatial location of cells in structural micrographs, or the assumption that the overlap in data from different specimens indicates anatomical invariance. But the purpose of this research program was not to test a specific theory that was based on such assumptions, but to provide a *prototype* of and *inferential norms* for reasoning about metazoan neural organization or the individual variability of its connectional patterns. Only such further experiments—made possible by the information provided in the wiring diagram—could lead to results which question the principles used to construct the diagram in the first place.

The acknowledgment that wiring diagrams of neuronal connectivity represent a revisable standard for further research is equivalently stressed by neuroscientists studying the human brain. Based on the principle that “individual brain regions maintain individual connection profiles”, Sporns et al. (2005, p. 0247) proposed that the structural mapping of axonal projections via diffusion-weighted imaging, combined with functional connectivity measurements via fMRI, MEG and EEG, would provide “a first draft of the human connectome” at the macroscopic scale. The research program proposed by Bohland et al. (2009) amends such a draft by mapping mesoscopic connectivity patterns with high-throughput neuronal tracer, neurotropic virus and viral vector methods in the mouse, targeted and standardized connectivity experiments in the macaque, and the application of these results to a technologically improved histological mapping of human neuroanatomy. On a conceptual level, Swanson and Bota (2010) provide a consistent, multi-scale classification system required for the neuroinformatics analysis of structural connectivity data, which nevertheless allows for empirical revision and the comparison of competing classification methods.

What the examples above indicate is that model-building and exploratory experimentation are not mutually exclusive activities, but that scientific practice is instead often characterized by *iteratively* switching between various modes of research. As a result of the assumption that “the mind is what the brain does” and a narrow focus on hypothesis testing, iteration in neuroscience has so far only been discussed as the repetitive reassessment of knowledge claims about the human brain through the continuous refinement of cognitive hypotheses (Kosslyn, 1999). What is therefore missing is how *methodological* iteration, i.e., the alternation between exploratory experiments, hypothesis-testing, technological development and question-generation *initiates* and *equips* the reassessment of knowledge claims (Elliott, 2012). Human brain mapping is not yet in the position to enter this iterative mode of research, because the missing knowledge about the mesoscale requires further exploratory experiments that establish more adequate neuroscientific concepts. The outline of parallel research strategies and wiring diagram projects at different scales, however, suggests that exploratory research should neither be seen as a *separate*, or even preliminary stage of scientific inquiry followed by hypothesis-testing and theory-building, but rather as “profoundly important for *ongoing* scientific activity” (O’Malley et al., 2009, p. 613).

What deserves special attention is that the large-scale connectomics projects substantially diverge in their definition of the mesoscopic scale. While Sporns et al. (2005) identify cortical minicolumns as the basic mesoscopic organizational unit, Bohland et al. (2009) postulate invariant connectivity patterns that would contain cortical columns (which are composed of many minicolumns) as “microcircuitry on a finer scale” (ibid., p. 3). Swanson and Bota (2010), in turn, view mesoscopic connectivity as the stereotypic possibilities of certain neurons (e.g., spinal nerve ganglion cells) to act, but also admit a variety of valid but competing classification methods of nervous cell types. Here, a convergence or mutual amendment seems to be less likely. Many columnar structures in the cortex, for instance, show heterogeneous response properties, vary in their presence within or between species, and do not decompose into a precise number of mini-columns. Such discrepancies have led skeptical commentators to conclude that the vertical cell bands found in Nissl-stained sections represent a functionally insignificant byproduct of ontogenetic development (so-called ontogenetic columns, cf. Horton and Adams, 2005). Current scholars have in turn suggested to either dynamically reconceive the column concept in terms of distributed connectivity networks or to replace it with successor concepts such as the “canonical microcircuit” (da Costa and Martin, 2010). Of course, such controversies over the conceptual foundations of neuronal assemblies or the issue of cross-classification of neuron types do not make the efforts to establish wiring diagrams obsolete. They rather show again that these diagrams will only lead to a more comprehensive understanding of human brain function when they are *combined* with the search for descriptively adequate, non-cognitive concepts via mesoscopic exploratory experiments.

It should be finally remarked that although connectomics research seems to date the most promising candidate to meet the brain “on its own terms”, its aims are often equally framed under the assumption that “the mind is what the brain does”. Sporns et al. (2005, p. 0248), for instance, hypothesize that the structural connectome places constraints on brain dynamics and “shapes the operations and processes of human cognition” while Bohland et al. (2009, p. 3) write that mesoscopic connectivity patterns will “aid our understanding of specific mental functions”. Although such formulations may be tentative, they overlook a crucial feature of the exploratory establishment of wiring diagrams. Since the terms “function” and “cognition” are not co-extensive, a complete mapping of structural connectivity could indicate functionally significant patterns of brain connectivity that are unintelligible from our current cognitive outlook on human brain function. A comparison with the Human Genome project is particularly instructive here: while only 1% of the genomic sequences code for protein—the initial definition of “gene”—mapping the other 99% revealed entities (e.g., micro-RNAs, distal enhancers) that turned out to be more crucial to genetic understanding than initially anticipated (Lichtman and Sanes, 2008). In light of the conceptual indeterminacies and required technological development at the mesoscopic scale, it is more than likely that a further exploration of the human brain will disclose new entities whose function cannot be captured with our current cognitive concepts. By temporarily suspending the use of cognitive concepts altogether, connectomics could therefore achieve its full potential for tackling how the human brain works.

## Conclusion

This paper proposed an alternative philosophical understanding of human brain mapping according to which neuroscientists do not have to presuppose that “the mind is what the brain does” in order to investigate human brain function. By stressing the methodological autonomy of neuroscience from psychology, the framework can capture the non-cognitive aspects of connectomics methods which have not been substantially discussed in philosophy of neuroscience yet. By systematically applying the philosophical and historical literature on exploratory experiments to neuroscience, the paper also showed how this framework can be applied in neuroscientific practice, if researchers investigate human brain organization without testing cognitive hypotheses or using cognitive concepts. The discussion of such exploratory research strategies revealed the following desiderata for further empirical research: based on the possible independence of neuroscientific laws from psychological generalizations, researchers should *provisionally extend* the concept of “functional connectivity” to the mesoscopic scale. The parallel experimental *identification of neural entities* irrespective of their causal roles for cognitive functions, combined with the *technological development* of mesoscopic measurement and intervention techniques, could then determine whether the provisional extension of “functional connectivity” has been warranted. In the meantime, further macroscopic aspects of neural connectivity can be established by the exploratory use of wide whole-brain imaging instruments. The validity and integration of such system-level results will depend on which organizational units can be experimentally established at the mesoscopic scale. Note that in exploratory research, the experimental conditions are themselves the criteria for identifying such units (as opposed to the functional identification of brain regions via task responses in hypothesis-driven cognitive neuroimaging studies). Currently, the experimental designs of functional connectivity studies do not provide necessary conditions for identifying large scale networks (cf. section “Exploring the brain with new concepts”) nor do the different clustering algorithms in such studies equally account for gradually changing connectivity profiles (cf. Haueis, 2012). Therefore, the purpose of this paper was not to *define* the criteria for identifying neural entities non-cognitively, but to show *how* brain researchers could establish such criteria if they would implement exploratory research strategies such as the ones outlined above.

It should be mentioned again that the notion of “meeting the brain on its own terms” is not intended to be an argument to disprove that the “mind is what the brain does”. It was rather meant to show that this assumption is not without alternatives, given that discovery-based connectomics approaches have the potential to reveal non-cognitive working principles of human brain organization. Applied to the mesoscopic scale, this potential can be fulfilled independently of our current cognitive outlook on human brain function, to which connectivity researchers commonly relate their results. Once further exploration leads to new concepts and principles that capture mesoscopic brain organization, researchers could re-assess our current cognitive concepts, and whether they are still adequate to capture this more complex picture of how the human brain works.

## Conflict of interest statement

The author declares that the research was conducted in the absence of any commercial or financial relationships that could be construed as a potential conflict of interest.

## Footnotes

^1^The perspective of this paper is to develop an alternative philosophical understanding of human brain mapping. The discussion of particular studies is therefore not meant to question their empirical validity, but to constructively point out the potential and issues of cognitive approaches that are hypothesis-driven, and discovery-based connectomics studies. By cognitive approaches, I would also broadly include studies of social and affective neuroscience, as long as they are attempting to functionally localize capacities like empathy, romantic love or various emotions in the human brain.

^2^Various philosophers of neuroscience have interpreted the supposed identity between mental capacities and brain activity in the above statement differently, as implying a reduction of cognitive processes to neural events (Bickle, 2003), as a heuristic theory of identity between cognitive operations and neural mechanisms (Bechtel, 2008), or as expressing that the correct theory of the mind will also be the correct theory of the brain (Roskies, 2010). Craver (2007) argues that psychological phenomena are constituted by the neural mechanisms that explain them, but holds open the possibility that the current cognitive conceptual framework “could be ill-matched to the mechanistic structures of the brain” (ibid., p. 124).

^3^Independence is therefore a related but stronger demand than “functional triangulation”, by which circularity is supposed to be overcome on the basis of between-study comparisons of tasks, designs and techniques within the cognitive neuroimaging approach (Roskies, 2010). Such checks are important to ensure the quality, but are insufficient to assess the adequacy of functional localization studies for investigating human brain organization (see the discussion of scale and inhibitory-excitatory microcircuits below, which Roskies mentions as well).

^4^Stressing the potential incompatibility of connectomic and cognitive neuroscientific results adds another dynamic component to Wimsatt’s account of detecting the properties of a system by various means of access (cf. Wimsatt, 1994). Wimsatt takes the initial convergence (in this case: the convergence of connectomics results with results from other histological or imaging methods) to establish both how robust a property and how reliable the technique detecting it is. Once robustness and reliability are established, divergent results of different methods can indicate aspects of the system unknown so far. I would add that discovering such new aspects can also retroactively change the relations among previous evidence in a way that former assumptions about the system need to be given up (for a case study of this temporal process in molecular biology, see Rheinberger, 1997).

^5^Such a parallel does not imply that generalizations in neuroscience need to possess the same invariance as physical or chemical laws. General statements about brain organization have to accommodate for different constraints (evolutionary and individual variability, stochastic occurrence of neuronal events) and serve different purposes (such as integration of molecular, cellular and brainwide aspects of neural mechanisms) than physical or chemical laws, (see also Lange, 2000a).

^6^Hacking (1983) argues that this independence is required to regard experimental observations as theory-free in the relevant sense (and thus “determinate” in Bechtel’s sense). Moreover, since the magnetic properties of the MRI contrast are well known (Huettel et al., 2004), Bechtel’s comparison of fMRI to the unknown chemical properties in Golgi staining seems to be no parallel at all.

^7^Recent advances in optogenetics (i.e., the insertion of light-sensitive genes which induce cellular activity) may address some of these issues. In a recent study, Lee et al. (2010) optogenetically induced neuronal spiking in genetically identified cell types of anesthetized rats, while carefully controlling for influencing factors at the system level (e.g., ventilation levels and frequencies, rectal temperature cf. ibid., 788). Whether this method resolves causal ambiguity remains controversial, however, since the final neuronal response measured is the net outcome of all inhibitory and excitatory activities of a cortical microcircuit (see the discussion in Logothetis, 2010). Additionally, since an equivalently tight system control of humans seems unlikely, optogenetic methods may not be directly transferrable to the human case.

^8^To put it in an apt metaphor one neuroscientist once suggested in a personal correspondence with me: Everything in between the function of single neurons and the whole brain constitutes the “Bermuda triangle of the neurosciences”.

^9^Since every scientific instrument possesses inherent limitations (noise, artifacts, limits in resolution etc.), increasing control or descreasing ambiguity should be seen as the practical norms against which technological progress is evaluated. Invasive electrocorticography (ECoG), for instance, could be developed into a “mesoscope” if the precision of the electrodes (currently measuring *ca*. 500,000 neurons) would be increased by an order of magnitude through further technological development. Even with such an advance, however, ECoG would remain practically limited to neurosurgical patients, and may still possess the ambiguity of LFP signals found by microelectrode recordings (see Ojemann et al., 2013 for a review of ECoG, BOLD and LFP measurements in epilepsy patients).
